# Human-in-the-loop error detection in an object organization task with a social robot

**DOI:** 10.3389/frobt.2024.1356827

**Published:** 2024-04-16

**Authors:** Helena Anna Frijns, Matthias Hirschmanner, Barbara Sienkiewicz, Peter Hönig, Bipin Indurkhya, Markus Vincze

**Affiliations:** ^1^ Institute of Management Science, TU Wien, Vienna, Austria; ^2^ Automation and Control Institute, TU Wien, Vienna, Austria; ^3^ Cognitive Science Department, Jagiellonian University, Krakow, Poland

**Keywords:** human-robot interaction, multimodal interfaces, transparency, failure, errors, human-robot interaction design

## Abstract

In human-robot collaboration, failures are bound to occur. A thorough understanding of potential errors is necessary so that robotic system designers can develop systems that remedy failure cases. In this work, we study failures that occur when participants interact with a working system and focus especially on errors in a robotic system’s knowledge base of which the system is not aware. A human interaction partner can be part of the error detection process if they are given insight into the robot’s knowledge and decision-making process. We investigate different communication modalities and the design of shared task representations in a joint human-robot object organization task. We conducted a user study (*N* = 31) in which the participants showed a Pepper robot how to organize objects, and the robot communicated the learned object configuration to the participants by means of speech, visualization, or a combination of speech and visualization. The multimodal, combined condition was preferred by 23 participants, followed by seven participants preferring the visualization. Based on the interviews, the errors that occurred, and the object configurations generated by the participants, we conclude that participants tend to test the system’s limitations by making the task more complex, which provokes errors. This trial-and-error behavior has a productive purpose and demonstrates that failures occur that arise from the combination of robot capabilities, the user’s understanding and actions, and interaction in the environment. Moreover, it demonstrates that failure can have a productive purpose in establishing better user mental models of the technology.

## 1 Introduction

In Human-Robot Interaction (HRI) scenarios, failure situations invariably arise despite the best efforts of system designers. These failures can be caused by multiple factors, such as sensor noise or misinterpreted user input. A human interaction partner may be able to remedy errors. Existing work dealing with failure with the help of a human interaction partner often focuses on the communication of robot failures that the robotic system is assumed to be aware of. For example, in the works by Van Waveren et al. (2022), Das et al. (2021), Das and Chernova (2021), failure is modeled as a failure state during plan execution by a robot, for instance, getting stuck on a carpet while navigating (Van Waveren et al., 2022), or being unable to pick up an object as it is located underneath another one (Das and Chernova, 2021). However, there is a gap in the literature when it comes to designing robot communication that makes it possible for the user to spot errors that went undetected by the system itself.

Our general focus is on situations where there is an error in the system’s knowledge base that the system is unaware of but that a user may notice. This requires that a human user is aware of the current state of knowledge of the robotic interaction partner. When the robot’s knowledge is conveyed to a human user by means of a shared task representation, this representation can subsequently be inspected, verified, and corrected by the user, if necessary.

We consider the specific scenario of a robot at a user’s home with the task of tidying up. It needs to know where each object is supposed to go, i.e., this user’s personal preferences. When building a system for performing household tasks, it is necessary to consider how to represent the robot’s knowledge to a human user and what types of failures can be expected to occur. In our study, a human shows a robot where to place certain household objects on a shelf.

Our work has two main aims. Our first aim is to find out how to support human-in-the-loop error detection in an object organization task with a robot through the communication of the robot’s knowledge base. Our second aim is to investigate if and how the errors that occur with a functional system fit into the current understanding of failure in HRI scenarios.

**FIGURE 1:**
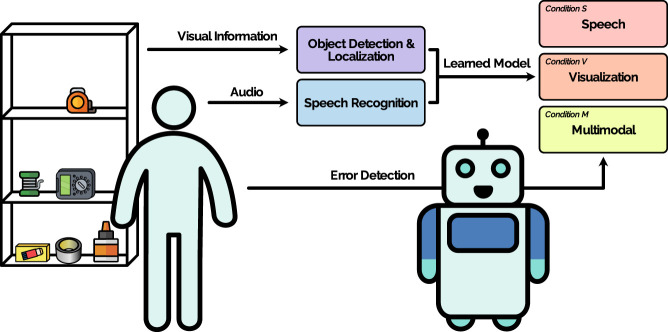
Overview of the system, setting, and study conditions. The task for the user is to arrange objects on a shelf to teach the robot the preferred configuration. The robot detects the position of the objects (*Object Detection and Localization*) and communicates the learned object configuration to the user. We compare three different types of output: *Speech*, *Visualization*, and a combination of speech and visualization (*Multimodal*). The user checks the output of the robot to detect errors (*Error detection*).

Towards the first aim of supporting human-in-the-loop error detection, we developed representations of a robot’s knowledge base for an object organization scenario. We conducted a user study in which the robot communicates a representation of its knowledge base (spatial locations of the organized object) to the user. In the study, we asked the participants to organize common household objects on a shelf. The robot then communicated its understanding to the user in one of three conditions; using 1) a visualization on its tablet, 2) speech, or 3) a multimodal condition combining both speech and visualization. See Figure 1 for an overview. We compare different output modalities, as it is not self-evident which modality will be preferred due to the spatial nature of the task involving an embodied robotic platform and objects organized at different spatial locations. Speech has an advantage, as it allows a user to focus their visual attention on the configuration of objects, while listening to the robot verbally indicating the objects’ locations. A potential disadvantage is that speech may be perceived as slow, and thus, visual communication may be preferred. Combining the modalities may result in information overload and/or combine the disadvantages of both modalities. Hence, it is important to investigate this issue empirically. We analyzed the results to understand the advantages and disadvantages of different interaction modalities and errors that occur in such scenarios.

Our second aim is to investigate if the errors that occur in a study with a functional system fit current failure understanding in HRI. Often, pre-programmed, pre-determined or wizarded errors are used in studies on failure in HRI without studying other potential errors (see, e.g., Hamacher et al., 2016; Mirnig et al., 2017; Kontogiorgos et al., 2020a; Hald et al., 2021; Nesset et al., 2021). This means that the failures that occur in such experiments do not include potential user errors or errors that arise from interaction with the environment but rather focus on hypothesized robot failures. Working with a functional system that incorporates object detection allows for a more realistic investigation of errors that occur in service robotics (and in object organization tasks specifically) and to determine which representation design is adequate in a naturalistic task setup. In our study, we did not pre-plan the failure situations. We programmed the robot to go through the interaction script and perform an object detection routine fully automatically. As we used a functional object detection system in which participants were not constrained in the way they organized objects or interacted with the system, we were able to find out which types of errors actually occur. A subset of encountered failure cases arose from user curiosity regarding the capabilities of the system and do not fit into current failure taxonomies in HRI, which often assume a single source of failure. In the user study, we encountered failure cases that have a function in user learning. While these cases are failures in terms of not achieving the performance of the intended function or task, they do perform a productive purpose in terms of contributing to user learning or satisfying user curiosity. The cases we observed describe a type of failures that arises from a combination of user actions on the environment, user expectations, and robot capabilities/limitations, which extends current understanding of failure in HRI.

In line with the aims sketched above, our work is guided by the following research questions:

RQ1 How to support human-in-the-loop error detection in an object organization task with a robot?
RQ2 How do the failures that occur with a functional system fit into current understanding of failure in HRI?

The contributions of this paper are as follows:

1. We present proposals for the design of representations of robot knowledge of object arrangements to human users by means of speech and/or visualization (Sections 3.3, 3.4), towards *RQ1*;
2. We present findings from a user study regarding user preferences on communication of robot knowledge in a human-in-the-loop error detection task (Sections 5, 6.1), towards *RQ1*;
3. We introduce the concept of productive failure in HRI, which is not part of any existing HRI failure taxonomies (Section 6.2), towards *RQ2*;
4. We argue for an understanding of failure in HRI as an interconnected phenomenon that develops over time and that can involve humans, robots, other agents, objects acting in the environment, which extends the understanding of failure in HRI failure taxonomies beyond one that considers failures as having a single source of origin (Section 6.2), towards *RQ2.*

## 2 Related work

In this section, we discuss related work on multimodal and transparent interfaces for robotic systems in Section 2.1, in line with our first aim. In Section 2.2, we outline concepts of failure and failure taxonomies in HRI, in line with our second research objective.

### 2.1 Design of multimodal and transparent interfaces in HRI

Previous research has investigated the use of individual modalities in HRI scenarios, such as displaying facial expressions for giving feedback (Mirnig et al., 2014), comparing unimodal displays of emotion (Tsiourti et al., 2017), or investigating preferences regarding a person-following robot’s auditory feedback behavior (Olatunji et al., 2020). In a Human-Computer Interaction context, multimodal input has been argued to offer advantages such as supporting user preferences and user learning, and reducing cognitive load and user errors when fusing information from user input modes (Goodrich and Schultz, 2007; Dumas et al., 2009). Advantages of multiple output modalities for communication from system to the user may be providing information in complementary forms (Park et al., 2018) or improved inferring of past causal information (Han and Yanco, 2023). In HRI, there is a gap regarding research comparing multimodal with unimodal inputs/outputs. In our work, we compare user preferences regarding the use of visual and auditory modalities, and a combination of the two, for presenting study participants with the configuration of objects detected by a robot.

Research on information presentation in relation to object configurations in HRI includes work on disambiguation or grounding of human requests, for instance for the PR2 robot (Guadarrama et al., 2013). Sibirtseva et al. (2018) argue that the use of natural language can result in ambiguous requests, and propose that using visualization can aid with disambiguation. In their user study, participants verbally describe an object, and the system visually indicates the inferred object by means of a head-mounted device, projector, or screen. Doğan et al. (2023) propose the Grad-CAM RGB method, which aims to identify specific objects in RGB-D images for human-robot collaboration in unstructured environments. They draw bounding boxes to indicate image regions in response to textual queries. Das and Chernova (2021) investigate semantically descriptive explanations of objects in a scene to help end users identify reasons for robot failure to pick up an object.

Such information presentation of a robot’s perception and processing is a form of transparency. Transparency refers to information being provided by a system with the aim to give a human end user a better understanding of what the system is doing and why (Frijns et al., 2023). This can concern information regarding a robot’s internal processes, for example, inferred commands (Perlmutter et al., 2016), robot plan representation (Wortham et al., 2016), learned words for objects and verbs (Hirschmanner et al., 2021), or providing a rationale for robot decision-making (Nesset et al., 2021). Hence, human interaction partners can better assess the robot’s state and form more accurate expectations regarding its behavior. Several works have investigated the design of transparent interfaces in HRI. Wang et al. (2023) developed an interface that visualizes the decision-making process of a robotic system and allows for inspection and editing of the knowledge graph. They argue that such an interface can support the sensemaking of robot decision-making. Perlmutter et al. (2016) performed a user study in which they compared a baseline of transparency through speech, pointing and gaze to a combination of the baseline with visualization-based transparency on screen, and to a combination of the baseline with visualization in Virtual Reality. The scenario concerns object manipulation and surface cleaning by the PR2 robot in response to human commands. Visual transparency enhanced the accuracy of commands and required less time, and increased the number of observed pointing gestures by participants. The screen-based visualization condition was preferred by participants. Hirschmanner et al. (2021) investigated a language-learning scenario of a human tutor teaching a Pepper robot words for objects and actions. They compared different transparency strategies, namely, pointing and gaze by the robot to request information regarding particular objects, and visualization of learned words on Pepper’s tablet. The visualization led to a higher self-assessed knowledge of the system state as compared to pointing. Several studies looked at the connection between errors and transparency, notably also in connection to trust (Hald et al., 2021; Nesset et al., 2021). Hamacher et al. (2016) ran a user study in which the BERT2 robot carried out a kitchen task, either with or without an error, and with or without verbal communication when correcting the error. They found support for their hypotheses that higher transparency mitigates dissatisfaction in case of errors, and that participants prefer a more communicative system.

In contrast to works that investigate how to communicate robot *failure* (Das and Chernova, 2021; Das et al., 2021), we investigate how to communicate a robot’s *knowledge base* so that the user can inspect whether it is correct or a failure has in fact occurred. This is similar to work on transparency, e.g., by Hirschmanner et al. (2021), Perlmutter et al. (2016) who visualize recognized speech, objects, and actions and compare different ways of communicating. However, in our work, we explicitly focus on human-in-the-loop error detection.

### 2.2 Failure and errors in HRI: concepts and taxonomies

In the definition by Brooks (2017), a failure occurs at the level of the task or functionality that a robotic system is supposed to perform: *“A “failure” refers to a degraded state of ability which causes the behavior or service being performed by the system to deviate from the ideal, normal, or correct functionality”* (Brooks, 2017, p. 9). This definition of failure is used for the taxonomies by Zhang et al. (2023), Honig and Oron-Gilad (2018). Similarly, failure has been defined as *“an event that occurs when the delivered service deviates from correct service”* (Steinbauer, 2013, p. 345) or the *“inability of the robot or the equipment used with the robot to function normally”* (Carlson and Murphy, 2005, p. 423). In the works by Van Waveren et al. (2022), Das et al. (2021), Das and Chernova (2021), failures are conceptualized as actions in a plan that fail, which results in a failure or halting of the robotic system’s plan. Several authors describe a relation between failures, errors and faults. According to Brooks (2017), Carlson and Murphy (2005), Honig and Oron-Gilad (2018), faults may lead to errors, which in turn may lead to failures. Applying this description to the task in our study, we can describe an object that is missing from the knowledge base representation as a failure in the robot’s task to detect organized objects and convey its knowledge base to the human interaction partner. This can occur due to an object detection error (e.g., white glue bottle is not detected), which can be caused by the fault of overexposure of the camera image.

HRI failure taxonomies are categorizations of failures that occur in HRI scenarios. Although it is unlikely that all possible robot failures can be identified for mobile robots in changing environments combined with a wide variety of possible interactions (Honig and Oron-Gilad, 2018), several authors have proposed failure taxonomies that aim to classify failures. See Supplementary Table 1 for an overview of failure taxonomies in the HRI literature. Honig and Oron-Gilad (2018), Carlson and Murphy (2005) classify based on the source of failure. The taxonomy by Honig and Oron-Gilad (2018) distinguishes between technical and interaction failures. Human errors are a subcategory of interaction failures, and can be mistakes, slips, lapses, or deliberate violations, based on the work by Reason (2009). Carlson and Murphy (2005) propose a taxonomy of failures for mobile ground robots (Unmanned Ground Vehicles, UGVs). They categorize failures according to their source, and failures are divided into physical and human failures. Tolmeijer et al. (2020) classify trust-relevant failures in HRI based on a different choice, namely, if the action of breaking trust was by the system or the user, and if this agent was supposed to act this way. They identify the failure types of Design, System, Expectation and User.

Some authors argue that failure categorizations should be more human-centered. Tian et al. (2021) write that the majority of HRI research takes a robot-centric perspective on failure. They note that failure is not only based on robot capabilities but also on its alignment with the context and whether the robot’s behavior is socially aware. Zhang et al. (2023) argue that while existing typologies classify failures according to what technically caused the failure, users do not know what caused the failure on a technical level, but make an assessment based on the information they have. They propose an output-oriented typology of performance failures, classifying them into logic, semantic and syntax failures. They classify performance failures in HRI based on expected *versus* actual output. In their taxonomy of social errors in HRI, Tian and Oviatt (2021) classify according to *“five categories that humans adopt to perceive socio-affective competence and social relationships”* (Tian and Oviatt, 2021, p.14) (see Supplementary Table 2).

Existing failure taxonomies and conceptions of what failure entails influence study designs in HRI. For example, Kontogiorgos et al. (2020a), Kontogiorgos et al. (2020b) performed Wizard-of-Oz studies in which they based the failure types that a robot made on the failure taxonomy by Honig and Oron-Gilad (2018). This illustrates that the way failure is conceptualized in taxonomies and definitions is important for the studies that will be conducted and contribute to our knowledge of the topic of failure in HRI, as well as the mitigation strategies that are proposed for robotic systems that encounter failure situations. In our work, we investigate how the errors and failures we encountered in our user study fit in current understandings of failure in the HRI literature, and argue how this understanding should be extended. In contrast to existing taxonomies that classify failures based on their source, we argue that HRI understanding of failure should acknowledge failure cases that arise due to a combination of technical capabilities (and limitations), human understanding and behavior, and interaction with the environment and other agents and objects in the context. Moreover, in our study, we observe user actions that do not fit neatly into the way “human errors” are currently described in such taxonomies.

## 3 Designing a system that conveys detected objects to a user

Our goal was to compare different ways of communicating robot knowledge of object configurations. In the envisioned scenario, a study participant organizes objects at a particular location. The robot learns user preferences from observations. The robot stores the object locations and conveys the configurations back to the user, who checks if these are correct, so the robot can (hypothetically) organize the objects by itself later. We investigated different ways of communicating the stored object locations. The communication of the stored object locations thus functions as a shared task representation. The concept of shared task representations has been proposed as a way to reduce common ground uncertainty in human-robot joint activities (Clodic et al., 2017; Frijns et al., 2023). Common ground (Clark, 1996) refers to a set of mutual or shared beliefs held by the human(s) and robot(s) involved in the interaction. We investigated how to design shared task representations and which interaction modalities are suitable for human-in-the-loop error detection.

### 3.1 Object detection

For object detection, we integrated a YOLOv5 (Jocher et al., 2022) implementation with ROS Noetic. The model was trained to recognize 11 objects (tools and objects commonly found at a workshop, e.g., measuring tape and wood glue). We used the copy-paste data augmentation technique to generate a diverse dataset by pasting images on random backgrounds (Ghiasi et al., 2021). We recorded 100 images per object from multiple viewpoints. We applied the DINO-ViT algorithm (Amir et al., 2022) to generate object masks. After filtering corrupted masks due to reflections, 20–50 image-mask pairs were kept per object, which was sufficient to cover the full surface of each object. The masked objects were randomly pasted onto backgrounds from the COCO dataset (Lin et al., 2014), resulting in 50,000 images with more than 3,000 instances per class. YOLOv5 was trained using the parameters set as suggested by the original implementation of Jocher et al. (2022). From the 11 objects on which the detector was trained, we used the 6 objects with the most reliable object detection performance in the specific setup of the experimental study. We used OpenCV’s ArUco marker detection (OpenCV, 2023) to detect the location of the shelf to determine the object positions in relation to it. Object locations were detected in 2D image space.

**FIGURE 2:**
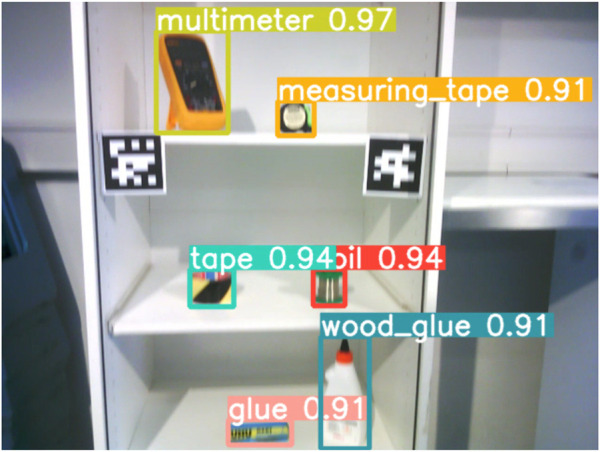
Example of the object detection using an inbuilt camera on the Pepper robot.

See Figure 2 for an example of the object detection running on the camera stream from one of Pepper’s cameras. During the experiment, the area in which the robot had to detect objects in the cupboard required the robot to move its head up and down to cover the area. The images from the camera stream had some motion blur. We combined the detection results from multiple camera images that covered the detection area, which took approximately 10 s. To give an idea of the system’s task performance, in the experiment there were 122 runs of the object detection routine, and in each of these runs 6 objects should be detected (732 in total). Excluding the 6 cases in which an object was fully hidden from view, we had 63 cases in which an object went undetected, yielding a performance of (1 − 63/726)∗100% ≈ 91.3% on the experiment task. The coil was detected 93.4% of the runs, the wood glue for 87.7%, the glue 84.4%, the tape 95.9%, the measuring tape 86.9% and the multimeter 100%. Note that in some of these cases, objects were partially hidden behind other objects. See Section 5.3.1 for more details.

### 3.2 Robot behavior

We programmed a Pepper robot to speak, perform gestures, and display information on its tablet with the Python SDK with NAOqi (SoftBank Robotics, 2023). The robot was programmed to execute slight motions to indicate that it is active, perform co-speech gesture and look at the participant (using face detection), which was implemented with the NAOqi API modules *ALBasicAwareness* and *ALAnimatedSpeech*. During object detection, the robot oriented itself to face the cupboard and moved its head up and down, verbally indicating that it was scanning the area. This behavior was intended to be both functional and communicative; the robot’s motion was used to collect image data of the full view of the cupboard and also expressed that object detection is being performed.

**FIGURE 3:**
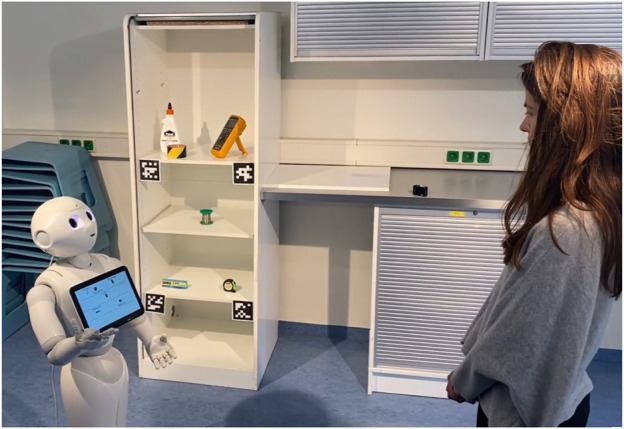
Robot turns to face the participant in conditions with visualization.

When the visualization was used, the robot turned to face the participant so that the left side of the tablet visually matched the left side of the cupboard from the participant’s perspective. This way, the participant did not need to cross-compare the visualization to the object configuration in the cupboard, see Figure 3.

### 3.3 Speech modality

The spoken description of object configurations by Pepper was based on research on descriptions of such configurations by native German speakers (Tenbrink et al., 2011; Tenbrink et al., 2016). Speakers tend to look for a salient object (a *relatum*), in relation to which they can describe the location of the target object. If a salient object is not available, they may begin with a reference region instead. Descriptions are usually sequential, following a continuous trajectory (Tenbrink et al., 2011; Tenbrink et al., 2016). We designed the speech condition as follows. We used two prepositions from Guadarrama et al. (2013), namely, *“to the left of”* and *“to the right of”*. A description was generated for each object. This is done on a per-area basis (e.g., considering those objects that are on a single shelf). When generating a description, the leftmost object was taken first and described using the approximate location of the object relative to the area, e.g., *“on the left of*,*” “somewhat to the left of*,*” “in the middle of*,*” “somewhat to the right of*,*” “on the right of”* + area name. Each next object in the area was described by referencing the previously described object plus the approximate location in the area. This generated descriptions such as: *“The wood glue is on the left of the top shelf. Next to the wood glue is the measuring tape, which is in the middle of the top shelf.”*

### 3.4 Visualization modality

**FIGURE 4:**
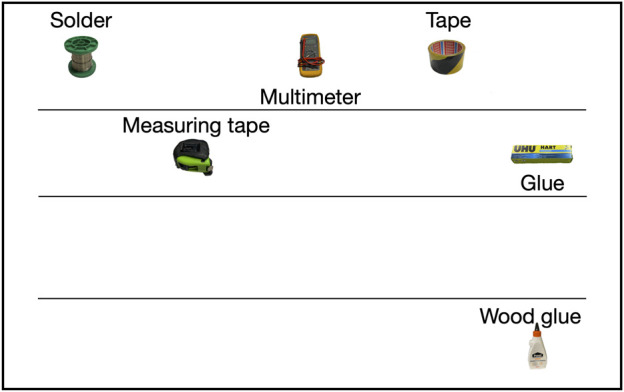
Example visualization in the *Visualization* condition.

Visualization options that were considered include an abstracted visualization with icons and text, a knowledge graph representation that closely matches the robot’s internal representation (as in, e.g., Wang et al., 2023), or a camera stream with a visual overlay (e.g., Perlmutter et al., 2016). In our task scenario, a camera stream representation would require either a static composition of multiple images from the video stream (which would result in a cluttered view that does not fit on the tablet in a way that individual objects can be distinguished); or it would need to change dynamically (which we expected would make it more difficult to keep track of the knowledge base). However, such a representation can be relevant for a follow-up study that involves troubleshooting when participants want to know why a particular object is wrongly detected or not detected. An internal graph representation was considered, but expected to result in a cluttered view as well. Therefore, we chose to represent the scene with an abstracted visualization, in which icons with text represent the objects. This representation provides an uncluttered representation of the scene that fits the tablet dimensions, as shown in Figure 4.

We used a JavaScript library (Lehni and Puckey, 2011) in a Choregraphe application package for the visualization, which could be dynamically adapted using Python commands. The visualization consisted of icons and names of the detected objects. Objects were displayed at a particular location, assigned to an area in a similar way as with speech (Section 3.3).

## 4 Study: human-in-the-loop error detection

A within-subjects study was conducted in which participants interacted with a Pepper robot in three conditions. The participant placed objects on a shelf to “teach” the robot their desired locations. After the objects were placed, the robot either communicated the object locations verbally [condition ***Speech (S)***], by means of a visualization on its tablet [condition ***Visualization (V)***, see Figure 4], or using both visualization and speech [condition ***Multimodal (M)***]. The communication of object configurations was done across communication channels that were additional to the robot’s nonverbal functional behavior (performing the object detection routine, Section 3.2), interactive behavior and verbal instructions to the participant.

We investigated the issue of human-in-the-loop error detection from different angles, namely, by considering participant behavior, participant interpretation of the system, failures that occurred with a functional object detection system, and participant preferences. The study research questions were as follows:

**RQ-Participant preference:** Which condition do participants prefer? Why?

**RQ-Task Load:** Do participants perceive a different workload between conditions?

**RQ-Error:** Do participants notice all errors? Is there a difference between conditions?

**RQ-Mental model:** How do participants construe the way the system works?

**RQ-Participant behavior:** Do participants use strategies to test the system, and when do they do so?

### 4.1 Study protocol

The experiment took place in a museum of science and technology in Vienna, Austria, in a separate room from the exhibition space. The interaction with the robot, questionnaires, and study procedure were in German. The study procedure and informed consent process were peer reviewed by the Ethics Committee at our university. The protocol included an explanation of the study, informed consent, interaction with the system in three conditions including completion of a survey form, and an exit interview.

**FIGURE 5:**
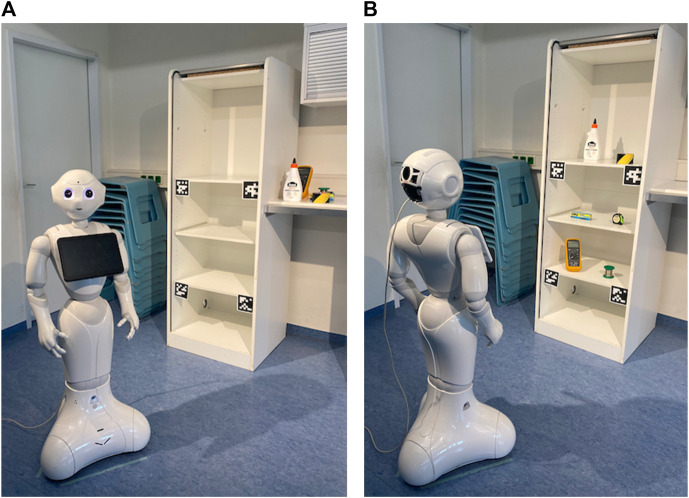
During the explanation **(A)**; after the participant arranged objects **(B)**.

First, participants were informed regarding the purpose of the study, data collection and storage, that they could opt out of the study at any time, and they were given researchers’ contact information. This information was included on informed consent and data consent forms that participants were asked to sign. Participants were asked to complete a short personal information questionnaire, and were introduced to the robot, how it worked, and its different sensors (Figure 5). The robot was programmed to indicate its sensors and describe their function, which was then repeated and explained by one of the researchers. This introduced the participant to the robot’s movement and enabled us to check that the robot’s speech volume was adequate for the participant. The researcher answered questions and explained the task.

In each interaction condition, the task for the participant was to place several objects on the shelves of a cupboard. First, the robot greeted the participant and announced it would scan the area. It then “scanned” the area by moving its head up and then down. Then, the robot asked the participant to arrange the objects in the cupboard and to indicate when the task was complete. The participant put the objects in the cupboard (wood glue, tape, measuring tape, a multimeter, a glue package, soldering tin) (Figure 5). When the participant said they were done (when the speech recognition detected the word *“fertig”/done*), the robot asked the participant to step aside. The robot performed the scanning motion again, during which object detection was running on the video stream of one of Pepper’s cameras. Depending on the condition, the robot spoke out the object locations (*Speech*), displayed them on the tablet (*Visualization*), or conveyed them by both speech and visualization (*Multimodal*). The robot asked the participant to confirm whether it was correct. If the participant confirmed, the robot thanked the participant, concluding the interaction. If the participant indicated it was incorrect, the robot announced it would scan the scene again from another perspective. It moved to the right and scanned the scene again, conveyed the object locations, and again asked the participant to confirm. If still incorrect, the robot stated it could not resolve the error. In either case, the robot thanked the participant, concluding the interaction. After each condition, participants completed a questionnaire. After interacting in all three conditions, participants were asked if they had time for an interview and were explained how the system worked.

The interaction with the robot was programmed to function fully automatically. As it was not the focus of our study, we chose to trigger the speech detection remotely if the built-in speech recognition did not function after the first two tries by the participant.

### 4.2 Measures

The interaction of participants with the robot was video and audio recorded. While the robot was performing object detection, a recording was made of its camera stream. An interaction log was kept automatically. Prior to the experiment, participants completed a questionnaire on personal information. After every condition, participants completed a questionnaire asking if the robot made mistakes regarding the locations of objects, and if yes, which. Other questions on the survey included if the robot made any other mistakes, how the robot communicated the information, and Raw NASA-TLX (Hart, 2006) translated to German (Flägel et al., 2019), to measure the users’ task load, which contains subscales for mental, physical and temporal demand, performance, effort, and frustration. After each participant interacted with the robot in all three conditions, they completed a final questionnaire on their preferred condition and the perceived condition order as a manipulation check. Questions in the structured exit interview included *Which version did you prefer and why? How did the robot learn the locations of the objects?* See Supplementary Table 3.

### 4.3 Pilot study

A pilot study was conducted at a museum of science and technology (*N* = 4, one woman, three men, age M = 35.25, SD = 6.02). The protocol as described in Section 4.1 was followed. One participant preferred condition *Multimodal*, stating it was the clearest, while three preferred *Speech* due to either not being able to see well on the screen, a concern that the visualization may not be clear enough in case the objects would be organized in a chaotic way, or feeling stressed with the combination and not always trusting the way it was displayed. After the pilot, minor changes were made to the questionnaire and the robot behavior. The robot’s behavior was changed so that it introduced itself and its sensors (instead of a researcher introducing the robot), which also allowed for checking if the robot’s speech volume was set at an adequate level for the participant prior to interaction in an experimental condition. The questionnaire was changed to ask the participant if the robot made an error in two different ways instead of once. After the revision, it asked whether the robot made an error both in terms of the object positions or if it made any other error.

### 4.4 Participants

For the main study, 33 participants were recruited in a museum of science and technology. Conditions were counterbalanced for 33 participants; the order of execution of counterbalanced conditions was randomized across all participants. This meant that five participants interacted in each of the condition orders VMS, SMV, and MSV, and six in each of the condition orders VSM, MVS, and SVM. Two of them did not pass the manipulation check: they did not correctly identify how the robot communicated the object positions on the survey forms after each interaction, nor on the final survey form.

In Section 5, we report on the data of the remaining 31 participants (16 men, 15 women). Their ages (M = 39.1 SD = 15.87) ranged from 17 (with parental consent) to 76. Nine participants indicated they had a robot at home (vacuum cleaner robot or LEGO). Nineteen participants stated having seen a robot, nine of whom had interacted with a robot, and two had programmed a robot (LEGO Mindstorms). Self-assessed computer programming experience was rated by 18 participants to be 1 (very inexperienced, i.e., have not programmed anything before), by 5 participants to be 2, by 1 participant to be 3, by 5 participants to be 4, and by 2 participants to be 5 (very experienced, i.e., professional programming knowledge).

## 5 Results

Thirty-one participants interacted with the system in all three conditions, yielding 93 interactions. The object detection routine would run once or twice per interaction, depending on the participants’ response to Pepper’s question whether the representation was correct after the first run of the object detection routine; if not, this routine would be performed again. This resulted in 122 runs of the object detection routine. Some interviews could not be used due to equipment failure. We decided to keep these participants in the analysis of the main study, since the equipment failure did not interfere with the interaction or the questionnaires. Additionally, the interview was designed to be voluntary (and presented as such to participants) to give participants the opportunity to clarify their questionnaire responses. The interview responses of twenty-four participants were transcribed and analyzed by coding the responses to the individual interview questions on preferred condition, the way the system works, and the way participants organized objects.

The interview analysis was conducted as follows. The first and the second author first familiarized themselves individually with the data, proposed a coding scheme, discussed the coding schemes together and then agreed on a coding scheme. See Supplementary Table 4 for an overview of the final coding scheme. Each of them individually coded the responses of the participants to the interview questions 2–5. For 89.7% of the 194 assigned codes the coders were in agreement (meaning that the two coders assigned the same code to the response). Coding differences were resolved by discussing each of the differences to arrive at a final joint coding.

### 5.1 Participant preference for interaction modality

To answer **RQ-Participant preference**, we looked at the questionnaire results and interview responses regarding the preferred condition. On the final questionnaire, 23 participants preferred condition *Multimodal*, 7 preferred condition *Visualization*, and one preferred condition *Speech*. A multinomial test (Robertson and Kaptein, 2016, p.142) yielded a *p*-value of *p* = 2.315∗10^–6^, thus we should reject the null hypothesis that each condition is preferred equally. An exploratory data analysis showed a difference in self-reported computer programming experience for participants who preferred the condition *Multimodal* (M = 1.57, SD = 1.16) and for participants who preferred the condition *Visualization* (M = 3.29, SD = 1.38). We performed multinomial logistic regression to check if “Programming Knowledge” can be used as a predictor variable for the preferred condition. The likelihood ratio test resulted in a *p* < 0.05 indicating that the model including programming knowledge as the predictor variable is significantly better than the null model. The coefficient for “Programming Experience” was 0.9052 (std err = 0.353, *z* = 2.566, *p* < 0.05), indicating that for every unit increase in “Programming Experience,” the log likelihood of participants preferring *Visualization* as compared to *Multimodal* increases by 0.9052. In a model comparing preference for *Visualization* to preference for condition *Multimodal* that included gender and age besides programming experience, the programming experience was the only predictor variable with *p* < 0.05.

In the interviews, participants stated a variety of reasons for preferring a specific condition. Reasons for preferring *Multimodal* included that it was a double confirmation regarding what the robot saw, it being clearest, having to make more of an effort to think along with speech-only or the risk of forgetting or mishearing with speech-only, being better able to compare it, the participant having a left-right weakness so seeing makes it easier, it reducing the chance of making mistakes, and the combination resulting in more humanlike communication. Reasons for preferring *Visualization* included that it is faster, being used to tablets, finding it easier to see rather than hear, and it being more effort to check if both visualization and speech were correct. A reason for preferring *Speech* included that seeing it on the tablet was too much information.

### 5.2 Task load

**FIGURE 6:**
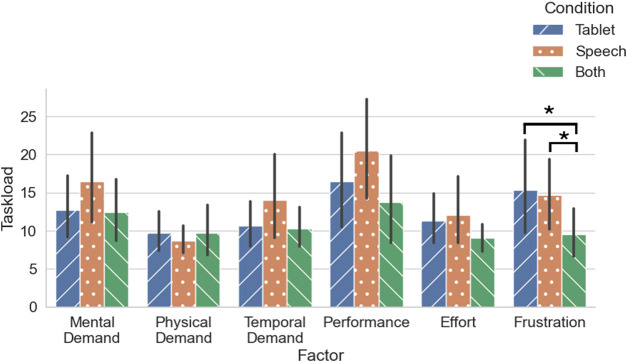
NASA-TLX subscale score averages of all participants (*N* = 31). *p < 0.01667.

The range for the 6 NASA-TLX subscales are from 0 (low) to 100 (high), and so are the total NASA-TLX scores that are calculated here by summing the subscale scores and dividing by 6. The total NASA-TLX means per condition (calculated using the scores of all participants) were as follows. For *Visualization*, the score was M = 12.69 (SD = 8.00), for *Speech* M = 14.41 (SD = 10.46) and for *Multimodal* M = 10.78 (SD = 6.43). The ratings were rather low for all three conditions; we observed a floor effect that makes it difficult to differentiate between groups. This tendency was not observed in the pilot study. See Figure 6 for an overview of subscale ratings averaged across all participants.

We performed Friedman tests (Robertson and Kaptein, 2016, p. 154) with Python (The SciPy community, 2008) for the total NASA-TLX scores and the subscales. We used the Friedman test, as the data is ordinal, and it is a repeated-measure (the study is within-subjects). We did a Bonferroni correction on the *α* level (0.05/7 = 0.007). The Friedman test for Frustration yielded a *p*-value of 0.0029 < 0.007. The other tests were not significant.

The mean scores for Frustration were as follows: for the *Visualization* condition M = 15.32 (SD = 18.21), for the *Speech* condition M = 14.68 (SD = 13.72), and for the *Both* condition M = 9.52 (SD = 9.07). As the Friedman test yielded a significant difference in Frustration among the different conditions, we did three Wilcoxon signed-rank pairwise comparisons for Frustration (following Robertson and Kaptein, 2016, p.155), applying a Bonferroni correction: 0.05/3 = 0.01667. We found no significant difference between *Visualization* and *Speech* (*Z* = 52.0, *p* = 0.6456). We found significant differences in the comparison of the *Speech* and *Multimodal* conditions (*Z* = 2.0, *p* = 0.0035 < 0.01667) and the *Visualization* and *Multimodal* conditions (*Z* = 3.5, *p* = 0.0030 < 0.01667). We can use $r=Z/\sqrt{N}$ to calculate the effect size for non-parametric tests (Robertson and Kaptein, 2016), which gives us a medium effect (0.36) for the comparison between the *Speech* and *Multimodal* condition and a large effect (0.63) for the comparison between the *Visualization* and the *Multimodal* condition.

To get an indication whether this may be due to one condition having more perceived error occurrences, we performed two Cochran’s Q tests (using Perktold et al., 2023). We performed a Cochran’s Q test to determine if there were differences between conditions whether participants reported errors or not, and a Cochran’s Q test if there were differences between conditions regarding whether there were any object detection errors or not. Interactions by participants with the system in which errors occurred were coded with a 0, and cases in which no error occurred were coded with a 1. We found no differences between conditions regarding the (perceived) occurrence of errors.

To answer **RQ-Task Load**, we found no significant difference between the total NASA-TLX scores. However, we did find differences for Frustration between the *Multimodal* condition and the *Speech* condition, and between the *Multimodal* and the *Visualization* condition.

### 5.3 Errors

To answer **RQ-Error**, we investigated whether participants noticed all errors and if this depended on the condition. This required identifying which errors occurred and comparing them to the errors that were reported by participants. We observed that participants were able to identify most errors in all conditions. In a few cases, participants did not notice errors, but these cases were too few to distinguish between conditions. We identify some modality-specific limitations. Further discussion is included in Section 6.1.

From the 93 interactions, in 41 cases the participant correctly identified that no errors occurred, while in 31 cases the participant correctly identified the errors that occurred. In 7 of those 31 cases, the participant responded to the robot that the representation was correct, but afterwards stated on the questionnaire that there was an error. In 12 cases, the participant stated there is an error but there was no object detection error *per se* (in those cases, only errors such as speech recognition errors occurred). In 9 cases, errors, inaccuracies or mismatches in the participant’s judgement were observed (Section 5.3.3).

#### 5.3.1 Object detection errors

We identified object detection errors (e.g., missing objects) by comparing the camera stream during object detection to the interaction log, which contained the results of the object detection. This was analyzed for each of the 122 times the object detection routine was performed. In 58 of these instances, one or more errors occurred. The main error concerned objects not being detected (69x), followed by detection of objects that were not there: in 7 cases, objects were detected that were in the data set but not in the set that participants were asked to organize (5x a marker, 2x pliers). Four object location errors occurred. The number of participants for whom one or more errors occurred was 11 (out of 31) during the first interaction, 11 for the second interaction, and 17 for the third and last interaction.

#### 5.3.2 Errors according to the study participants

Participants were asked to indicate on the survey form if the robot made (1) an error regarding the object locations or (2) any other errors, and if so, which ones, see Table 1. In addition to the object detection errors described above (Section 5.3.1), participants also reported speech recognition errors and social interaction errors (e.g., not turning towards the participant while speaking, long response times).

**TABLE 1:** Errors as indicated by participants on the survey form.

Error	Number of times mentioned on a survey form
Unspecified	3
Object detection errors	
Object(s) not detected/recognized/shown	20
Hidden object not detected	4
Could not show that an object was placed behind another	3
Wrong object mentioned	2
Additional object shown	2
Object position error	1
Interaction errors	
Speech recognition error	4
Next behavior triggered too soon before participant spoke	4
Speech was recognized too late	2
Response time too long	1
It confirmed twice	1
Robot did not turn itself to me	1

#### 5.3.3 Errors and inaccuracies in participant judgement

In 9 of 93 cases there was an error or inaccuracy in participant judgement based on the survey form results and their response to the robot (5x *Speech*, 2x *Visualization*, 2x *Multimodal*). However, participants were observed to make statements towards the researcher or display nonverbal behavior that is indicative of them noticing an error in some of these cases. We list the specific cases below:

•Twice (in the *Speech* condition) objects were not detected but the participant said there was no error (to the robot and on the form). In one case, three objects were missing. In the video footage, this participant addressed the researcher in a questioning way, saying that objects were missing, but that other objects the robot talked about were correct.
•One participant indicated twice (in the *Speech* and *Multimodal* conditions) that there was an error and indicated one missing object, but an additional object was also missing.
•Twice, an additional “unknown” object was detected that was not part of the objects participants were asked to organize, but that was part of the dataset (pliers, marker), without the participant indicating on the form or to the robot that there was an error. However, on the video data, one participant (*Visualization*) verbally remarked to the researcher that there was a marker, and one participant (*Speech*) initially nodded in response to each of the robot’s statements about object positions, but stopped nodding once the robot mentioned pliers, which could indicate that the participant noticed something was wrong.
•Twice (*Multimodal, Visualization*), a participant noted that the robot could not indicate that objects were placed behind each other, but in principle no object detection error occurred. In the *Multimodal* case, one object was missing from the representation during the second scan, which was not remarked on by the participant on the survey form; the participant remarked that the robot was not able to recognize that objects were placed behind each other or on top of each other.
•Once in the *Speech* condition, a participant stated that there was an error without specifying, but no error could be identified by us.

### 5.4 Mental model: participant interpretation of the object detection system

To answer **RQ-Mental model**, we coded the interview responses to the question *“How did the robot learn the positions of the objects?”* The participants made reference to scanning (12 participants), cameras or photography (7 participants), the objects being pre-programmed or the robot having an existing representation of the objects (6), the markers (5), seeing or eyes (3), the shape of the objects (3), with the text on the object (2), sensors (2), programming (1), the size of the object (1), the ultrasound and infrared sensors (1), or that it was done by a human (1).

In other words, participants explained how the robot detected objects by referring to the words used by the robot, to its behavior, what they could observe in the space, on the objects and in relation to the robot, and some made reference to its humanlike capabilities like seeing and reading. Participants had multiple sources of information available to them about the way the system worked: what the researchers told them at the start of the experiment regarding the robot’s sensors and the task, the words and behavior of the robot, what could be observed by the participants (e.g., ArUco markers on the cupboard), and the events during the trials. For example, the mention of scanning can be connected to the robot stating it was “scanning the area”. Some of the theories participants constructed were (partially) incorrect. For example, one participant stated: *“It obviously has a database of those objects, and it looks for those exact objects every time where they are. Last time I forgot the soldering tin outside. Then he didn’t realize that the soldering tin wasn’t there at all. So, obviously, he looks for exactly these objects every time on the shelf. And if one of them is not quite there, i.e., it’s not on the shelf, but it’s on the side, then he recognizes it as being on the shelf. Even if it is not on the shelf.”* This illustrates how accidental events during the trial influenced the participant’s understanding of the system. There was a technical failure during the trial: the soldering tin was detected as being on the shelf because the shelf area had been defined to be a bit wider than it actually was. So the soldering tin, which was forgotten by the participant and left next to one of the shelves, was also assigned to being on the shelf. This resulted in inaccurate understanding of the participant; the robot does not actively look for those exact objects.

### 5.5 Participant behavior: object arrangements

To answer **RQ-Participant behavior**, we analyzed both participants’ object organization strategies as well as their answers to the interview question that asked if they had a specific reason for the way they organized the objects. The strategies that were mentioned by participants in the interviews were coded, as were the strategies that were observed with regard to the object configurations.

In the interviews, several strategies were mentioned. Eleven participants mentioned testing the system in some way, for instance, by placing an object inside or behind another object, or rotating an object, to see if the system would still be able to detect the configuration. Ten participants mentioned organizing objects without any specific intention in mind. Five reported ordering objects by category (e.g., grouping adhesives or measurement tools). One other mentioned reason was making a clear, symmetrical arrangement. One participant stated: *“I thought I would give him easier tasks at the beginning and then more difficult ones. That’s just the way it is with a child. You just increase it.”* Another: *“So the first time I thought, nice and symmetrical, clear. The second time I made it a little harder, I think for him, because then I also put two things next to each other. And the third time, I turned the tube of glue on its side because I thought that if he only had the [brand name] as a pattern, he might stumble, and he did. It was mean, wasn’t it?”*

**FIGURE 7:**
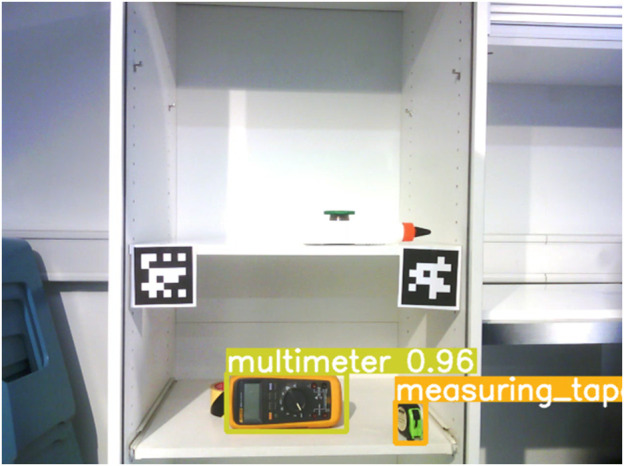
Example of an object configuration by a participant in which the objects are more difficult to detect by the system.

**FIGURE 8:**
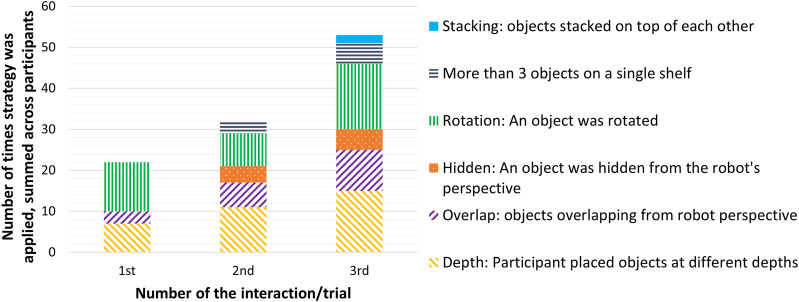
Number of times per interaction that participants applied object organization strategies with increased complexity, which made object detection more difficult. The strategies are summed up across all participants for the first, second and third time they interacted with the system. The frequency of complex strategies increased from the first to the third interaction (meaning that participants applied more strategies to “test the system” in the second trial as compared to the first, and applied even more strategies in the third and final trial). In other words, participants increasingly displayed testing behavior.

For analysis of the object organization strategies, the first and third author agreed on a classification and then coded screenshots of the organized cupboard (inter-rater agreement 92.1%, mismatches were resolved jointly). We distinguished several strategies that participants may have thought would make it more difficult for the robot to detect objects, which were also reflected on during the interviews. See Figure 7 for an example. The number of times this happened increased from the first interaction. Strategies identified included stacking an object on top of another, placing more than three objects on a shelf, rotating objects, hiding objects from the robot’s perspective or placing them in a way that they overlapped one another, and placing objects at different depths on the shelf. See Figure 8.

In the first interaction, an object was hidden behind another object zero times (0x), in the second interaction 4x, and in the third interaction 5x. Objects were placed in a way that one object overlapped another from the robot’s camera perspective 3x in the first interaction, 6x in the second interaction, and 10x in the third interaction. Placing more than three objects on a single shelf occurred 0x for the first interaction, 3x for the second, and 5x for the third. Stacking of objects only occurred two times, both in the third interaction. For rotation, the coding required distinguishing between canonical and non-canonical views, where canonical views would be those views that people find more typical or easier to recognize (Peters, 2001). Rotation of objects (a non-canonical view) remained more or less similar across interactions, with an object being rotated 12x for the first interaction, 8x for the second, and 16x for the third interaction (excluding measuring tape as it lacks a clear orientation).

## 6 Discussion

In this section, we discuss our findings regarding human-in-the-loop error detection to answer *RQ1* (Section 6.1) and how the observed failure cases align with current HRI understanding of failure to answer *RQ2* (Section 6.2) in line with the research objectives stated in the Introduction.

### 6.1 Supporting human-in-the-loop error detection

In this section, we discuss how to support human-in-the-loop error detection in an object organization task with a robot. Most participants (71.9%) preferred to get the representation of the robot’s knowledge both as a visualization and through speech, as this was a double confirmation. Moreover, the NASA-TLX subscale score for *Frustration* was lower in the *Multimodal* condition as compared to the single-modality conditions. As participants correctly identified whether the robot made an object detection error or not in 90.3% of cases (Section 5.3), this shows that all three conditions can support error detection by users. However, modality-specific advantages and limitations exist, as described below.

Regarding advantages and limitations of the visual modality, the persistent nature of the visual representation makes it easier to keep track, compared to only speech, as reported in participant interviews. An advantage of the *Visualization* is that it is seen as faster by some, while disadvantages are that it requires being able to see well and its limitations for representing complex scenes. In the visualization condition, it is important to be able to handle all possible object configurations, as some visualization-specific errors were reported. The robot that was used in the experiment was the Pepper robot, which has a built-in tablet. One solution to transfer our findings to other (humanoid) robots that do not have an in-built screen is by developing a smartphone or tablet app that performs a similar function.

Regarding the auditory modality, the speech representation resulted in uncertainty in some cases. Once in the *Speech* condition, a participant wrote on the form that they were unsure whether an object was missing (this was counted as reporting an error). As described in Section 5.3.3, in two cases in the Speech condition, objects were not detected but the participant said there was no error (to the robot and on the form). In one case in the Speech condition, the appearance of unknown objects was not remarked on to the robot or on the survey form. This suggests some errors were missed or participants were unsure due to the representation in the *Speech* condition not being persistent. The response of the participant for whom three objects were missing arose due to the nature of the representation; asking whether a speech-only representation is correct has the risk of the participant confirming its correctness, when the representation only partially covers the object arrangement. Uttering one true statement can be interpreted as the representation being correct, even if there is missing information; if that statement is correct, the missing information does not make the representation incorrect. For example, when the robot only mentions one of the objects at the correct location, this can be seen as a correct statement even if other objects are not mentioned. However, this may change if participants are more motivated to ensure the full representation is correct. With visualization, on the other hand, there may be more of an expectation that the representation completely matches the scene when asked to confirm its correctness.

An exploratory data analysis indicated that participants with higher self-reported computer programming experience were more likely to prefer the visualization-only condition as compared to a combination of visualization and speech. This indicates that novice users may prefer both interaction modalities while advanced users who are familiar with the system might prefer visualization only. We assume that the reason for this is that participants with more computer programming experience are more familiar with tablets and data visualizations on screens. Our results suggest to communicate information using both visualization and speech when a user starts interacting with a device and to offer the possibility to disable one of these at a later point. In our interviews, the multimodal condition was preferred as it was a double confirmation, which enhances certainty. Starting with both modalities would also be preferable in terms of accessibility. Speech can be helpful in case a user does not see well, and visualization in case a user has difficulty hearing. This should be considered in the design of robot embodiments; not all robots intended for human interaction include a built-in screen. A built-in screen or a connection to an external tablet allows for supporting human-robot joint activities with a visual shared representation.

A general observation for experiments in which participants are asked to detect errors is that participants should be asked if there are errors in several different ways, as they may respond differently to a robot than what they report on a form or to a researcher. Moreover, participants may have difficulty noticing additional errors after they have noticed an error. As described in Section 5.3.3, in three cases multiple errors occurred but the participant only reported on one of the errors. This could indicate that some participants may have focused on one error and did not pay attention to other potential errors that could have occurred, once they encountered an error.

### 6.2 How do the observed failure cases align with HRI understanding of failure?

In this section, we describe how the errors that occurred in the user study fit into current HRI failure taxonomies and understanding of failure in HRI. When looking at the errors from a technical perspective in Section 5.3.1, the resulting failures (objects being missing as they were not detected) can be described as software failures according to, e.g., the taxonomy by Honig and Oron-Gilad (2018). However, when we take errors reported by participants and participant behavior into account, a more complex picture emerges of the failures that occurred. In line with the calls by Tian and Oviatt (2021), Zhang et al. (2023), a more human-centered perspective on errors comes to the fore. The interaction errors according to the study participants as described in Table 1, namely, speech recognition errors, next behaviors triggered too soon, speech being recognized too late, long response times, confirming twice, and the robot not turning itself to the participant can be classified as social norm violations in the human-robot failure taxonomy (Honig and Oron-Gilad, 2018), or as social errors according to the taxonomy of social errors in HRI (Tian and Oviatt, 2021). However, adding social norm violations to a taxonomy is not enough to adequately describe all potential failure situations.

During our main study, we observed participant behavior that made the object detection more difficult (Section 5.5). This behavior was seen to increase as the interactions progressed. This may also have contributed to the increase in errors between the last and the first interaction (Section 5.3). Such behavior could be interpreted as the participants having a mental model of how the object detection works, forming a question in their mind (e.g., *“will the robot recognize the object if I hide it?”*), and then testing it in subsequent interactions. Participants also reported that they were testing the system (Section 5.5). Errors that were mentioned on the survey forms (Section 5.3.2) included that “hidden objects” were not detected. This shows that a subcategory of “human errors” exists that arise due to the human uncertainty about the potential effects of their actions (and neither due to deliberately acting in a way that sabotages the system, nor due to acting without any intention whatsoever). It also shows that errors exist that arise from a combination of technical and system design limitations, participant behavior, participant expectations and understanding of the system, and the configuration of objects in the environment. This makes it difficult to classify such failures according to the source of failure, which is the foundation of several failure taxonomies (Honig and Oron-Gilad (2018); Carlson and Murphy (2005); see Section 2.2).

Human errors in the taxonomy by Honig and Oron-Gilad (2018) include mistakes, slips, lapses, and deliberate violations, which are described as *“intentional illegitimate actions (e.g., directing the robot to run into a wall)”* (Tian and Oviatt, 2021, p.3). We argue that the testing behavior is a deliberate action, but it is not a deliberate violation *per se*; it is not an action that has the sole aim to make the system fail but rather to find out if a particular situation will result in a technical failure. This is desirable user behavior in low-risk situations, as it will improve user understanding of the way the system works. If a failure occurs, it is the result of an intentional action that serves a productive purpose, e.g., improving the user’s understanding of the system. This is related to the concept of trial-and-error behavior and its importance for user learning (Cakmak and Takayama, 2014). We argue that the opportunity for playful trial-and-error behavior, or productive failure, is something that should be considered in systems design and failure understanding in HRI. This concept is not a part of the current HRI failure taxonomies (e.g., Honig and Oron-Gilad, 2018; Tian and Oviatt, 2021). Usually, learning is not explicitly considered in HRI failure taxonomies. Learning mistakes are considered in taxonomies in other domains such as e-learning (Priem, 2010), where e-learning errors have been construed as potentially harmful by some authors, as it may lead to low computer self-efficacy or computer anxiety, while others construe error in a more positive light and see it as a part of the learning process. In low-risk HRI scenarios, we argue that errors in the context of trial-and-error behavior perform a positive role for user learning.

User learning to establish a more accurate mental model of the way the technology functions is especially relevant in the HRI context. The disconnect between people’s estimates of robot perception and reasoning capabilities and the robot’s actual capabilities has been referred to as *mental model discrepancy* (Perlmutter et al., 2016), asymmetry in perception (Frijns et al., 2023), and the perceptual belief problem in HRI (Thellman and Ziemke, 2020). As we observed that the way the robot’s behavior was designed impacted participant understanding of the way the system works (Section 5.4), we argue that interaction design can scaffold trial-and-error behavior and the development of a correct mental model of system function. To facilitate the user in gradually developing a more accurate model of the robotic system, it is important that the robot gives appropriate behavioral cues. Similarly, Tolmeijer et al. (2020) mention training and interaction design as potential failure mitigation strategies when a trust violation occurs. Interaction design can support user learning during interactions with a robotic system, where human interaction partners gradually develop a better understanding and expectations of the robotic system. For instance, this can be done through initial interactions in which object detection limitations are demonstrated, or by providing sensor information when the user detects an error.

## 7 Limitations of the study

Our study does not address long-term effects, as the participants were asked to organize the objects only three times. Moreover, the realism of the scenario was limited. The shelf was initially empty, and the participants were asked to place a limited set of objects on the shelf. In a more realistic setup, the cupboard may already contain some objects. In such situations, it makes sense to display only newly added objects. Another limitation was that the robot was not capable of performing object manipulation. This may have impacted the participants’ motivation to ensure that the robot correctly detected the objects. Honig and Oron-Gilad (2018) discuss motivation as an important aspect for solving failures and mention the mitigation strategy of setting expectations regarding potential errors. Fewer errors may occur if participants have a higher level of motivation, if they are given more specific instructions for object organization, or given insight into the robot’s perceptual processing. A different experimental setup, in which participants would be required to complete the task as fast as possible, may have led to a situation in which participants would not display trial-and-error behavior. The low task complexity and missing robot manipulation capabilities may have contributed to the observed floor effect for task load scores. Future work should investigate if higher task complexity will lead to more pronounced differences between conditions. As we ran the experiment with visitors to a museum of science and technology, there were regularly other people present in the room, e.g., friends and relatives. This could have influenced the participants’ behavior, for which the video data may be analyzed (see, e.g., Giuliani et al., 2015). Moreover, that the study took place in a museum of science and technology may also have contributed to the participants’ motivation to test the system’s capabilities and learn more about it, or this may have resulted in selection of participants with an interest in technology. Advantages of the study location were that the study participant pool was balanced in terms of gender and had a wide range in terms of participant age and programming experience. At the same time, the study was restricted to German speaking participants in Vienna, Austria. The number of participants was restricted due to practical constraints, which means the quantitative data gives limited information. However, the aim of the experiment was to gather qualitative data on interactions of participants with a system that produces non-wizarded errors.

## 8 Future work

Future work for human-in-the-loop error detection should include studying the efficacy of alternate visualizations. For example, the user could be shown the camera stream with bounding boxes around detected objects when the user detects a failure (different from the one chosen in our study, as explained in Section 3.4). The human interaction partner may also be offered follow-up actions such as repositioning the robot, an option to manually correct learned object locations, or to view sensor data. The system can be extended by incorporating capabilities to detect and represent more complex object configurations (using all spatial prepositions mentioned by Guadarrama et al., 2013), or object pose estimation to cover 3D. However, errors can still occur (e.g., due to occlusions). To deal with such limitations in technical systems, our work provides suggestions on how to support human-in-the-loop error detection. Future research needs to investigate how interaction design can support the user in developing a more accurate mental model of the robotic system.

## 9 Conclusion

In this work, we considered the problem of supporting human-in-the-loop error detection in an object organization task involving object detection functionality, a robotic system, and a human who organizes objects. In our study, we investigated if shared representations can support the participant’s error detection task. We evaluated efficacy of different output modalities for the design of shared representations that the end user can inspect to detect errors of which the system is not aware. We used a functional object detection system in our study, to investigate the types of errors that are likely to occur in practice.

We found that visualization, speech and a combined condition all supported error detection. For the speech-only condition, we observed most cases of uncertainty of participants. However, this condition also sufficiently supported error detection. Most participants preferred the combined condition. An exploratory analysis of our results suggests that users with more programming expertise prefer visualization; but this needs to be investigated further. We recommend using both speech and visualization to decrease uncertainty, especially at the start of the interaction, and offer the option to switch off one of these modalities as the interaction proceeds. Moreover, using both modalities has advantages in terms of accessibility. Our study shows that a visualization of the robot’s knowledge base, in addition to speech or by itself, is highly preferred in the context of a task that requires a human interaction partner of the robot’s internal state. Thus, a built-in screen or external tablet with a visualization of the knowledge base supports human-robot joint activities.

Secondly, as participants were able to freely interact with our system, we were able to observe failure cases that play a role in user learning. Participants were observed to gradually make the task more difficult for the robot, to test and better understand the system. Failures arose due to participants’ interpretation of and uncertainties regarding the robot’s behavior, their motivation to find out whether their interpretation is correct, participant actions on the environment and the robot’s subsequent perception of the environment. Such failure cases are not considered in current HRI failure taxonomies. When the user is sufficiently supported through the system’s interaction design, these types of failures are likely to positively contribute to the user’s understanding of the way the system works. Supporting trial-and-error behavior and designing robotic systems so that they help the user improve their mental model of the way the system works will help prevent failure in the long run. A related point we want to make, is that current failure taxonomies in HRI that categorize failures based on their source (e.g., a sensor or an action by a human) are likely to overlook how multiple sources can together lead to failure situations, or to miss certain failure cases altogether (e.g., the productive failure/trial-and-error behavior that we describe in this paper). We argue that failure in HRI should be understood as an interconnected phenomenon, where combinations of actions by different agents in the environment lead to failure.

## Data Availability

The raw data supporting the conclusion of this article will be made available by the authors, without undue reservation.
