# Supplementary material for: Human-in-the-loop error detection in an object organization task with a social robot
Source: Front Robot AI. 2024 Apr 16;11:1356827. doi: 10.3389/frobt.2024.1356827 (PMC11058786; doi:10.3389/frobt.2024.1356827)
Supplement: Supplementary file 1 [file Table4.pdf]

Table S4. Codes for analysis of interviews

Codes for analyzing responses to the questions *"Which version did you prefer?"*(Question 2) and *"Why?"* (Question 3)

Both, because...
- ... it is a double confirmation
- ... then you both see and hear it
- ... it is the most clear
- ... speech-only takes more effort
- ... then you can better compare/memorize it
- ... it is more humanlike to have both hearing and seeing in the communication
- ... I did not like the speaking away
- ... then I can't make mistakes
- ... I have a left-right weakness

Tablet, because...
- ... it is faster
- ... I am used to tablets
- ... conditions with speech are more effort

Speech, because...
- ... it is too much information to also see it on the tablet

---

Codes for analyzing responses to the question *"How did the robot learn the positions?"* (Question 4)

Participant made reference to:
- Scanning
- The objects were programmed in before or shown before, or it had existing representation of the objects (e.g., samples)
- Markers
- Camera/photo/photographically
- Seeing/with the eyes
- Sensors
- Program/programming
- Shape of object
- Determining the position of the shelves
- Size of object
- Ultrasound and infrared sensors
- Done by human
- With the text on the object

Codes for analyzing responses to the question *"Did you have a specific reason for the way you organized the objects?"* (Question 5)
- No reason
- Testing the system
- Symmetrical, clear arrangement
- Ordering objects by category (e.g., sticking, measuring, technical)
