# Supplementary material for: Human-in-the-loop error detection in an object organization task with a social robot
Source: Front Robot AI. 2024 Apr 16;11:1356827. doi: 10.3389/frobt.2024.1356827 (PMC11058786; doi:10.3389/frobt.2024.1356827)
Supplement: Supplementary file 2 [file Table1.pdf]

**Table S1.** Overview of different failure taxonomies in the HRI literature

| **Topic of taxonomy** | **Main categories** | Subcategory | Subtopic |
|---|---|---|---|
| **UGV failures**<br>Carlson and Murphy (2005) | Physical failures | Effector<br>Sensor<br>Control system<br>Power<br>Communications | |
| | Human failures | Design failures<br>Interaction failures | |
| **HRI failure taxonomy**<br>Honig and Oron-Gilad (2018) | Technical failures | Software failures | Design Failures<br>Communication Failures<br>Processing Failures |
| | | Hardware failures | Effectors<br>Sensors<br>Power<br>Control |
| | Interaction failures | Social norm violations | |
| | | Human errors | Mistakes<br>Slips<br>Lapses<br>Deliberate Violations |
| | | Environment & Other agents | Group-Level Judgement<br>Working Environment<br>Organizational flaws |
| **Faults in HRI/robotics**<br>Steinbauer (2013) | Interaction | Humans<br>Agents and Robots<br>Environment | |
| | Algorithms | Decision Making<br>Behavior Execution<br>Perception<br>Localization and Mapping | |
| | Software | Decision Making<br>Behavior Execution<br>Perception<br>Low Level | |
| | Hardware | Platform<br>Sensors<br>Manipulators<br>Controller | |
| **HRI failures**<br>Tolmeijer et al. (2020) | Design failure | | |
| | System failure | Hardware<br>Software | |
| | Expectation failure | Commission failure *(unexpected behavior by the robot)*<br>Omission failure *(the robot fails to act in accordance with user expectations)* | |
| | User failure | Intentional<br>Unintentional | |

## REFERENCES


Carlson, J. and Murphy, R. (2005). How UGVs physically fail in the field. *IEEE Transactions on Robotics* 21, 423–437. doi:10.1109/TRO.2004.838027

Honig, S. and Oron-Gilad, T. (2018). Understanding and resolving failures in human-robot interaction: Literature review and model development. *Frontiers in Psychology* 9, 861. doi:10.3389/fpsyg.2018.00861

Steinbauer, G. (2013). A survey about faults of robots used in RoboCup. In *RoboCup 2012: Robot Soccer World Cup XVI*, eds. X. Chen, P. Stone, L. E. Sucar, and T. Van Der Zant (Springer Berlin Heidelberg), vol. 7500. 344–355. doi:10.1007/978-3-642-39250-4_31

Tolmeijer, S., Weiss, A., Hanheide, M., Lindner, F., Powers, T. M., Dixon, C., et al. (2020). Taxonomy of trust-relevant failures and mitigation strategies. In *Proceedings of the 2020 ACM/IEEE International Conference on Human-Robot Interaction* (ACM), 3–12. doi:10.1145/3319502.3374793
