# Supplementary material for: Human-in-the-loop error detection in an object organization task with a social robot
Source: Front Robot AI. 2024 Apr 16;11:1356827. doi: 10.3389/frobt.2024.1356827 (PMC11058786; doi:10.3389/frobt.2024.1356827)
Supplement: Supplementary file 3 [file Table2.pdf]

Table S2. Overview of the taxonomy of social errors in HRI by Tian and Oviatt (2021) and typology by Zhang et al. (2023)

Topic of taxonomy	Categories
**Social errors in HRI**<br>Tian and Oviatt (2021)	Breach in empathetic and emotional reactions<br>Insufficient social skills<br>Misunderstanding the user<br>Insufficient communicative functions<br>Breach in collaboration and prosociality
**Output-oriented failure typology for HRI**<br>Zhang et al. (2023)	Logic failures *(involve that there is output that conforms with expectation, but the output is wrong)*<br>Semantic failures *(involve that the output format does not conform with expectation)*<br>Syntax failures *(involve that there is no output)*

REFERENCES

Tian, L. and Oviatt, S. (2021). A taxonomy of social errors in human-robot interaction. *ACM Transactions on Human-Robot Interaction* 10, 1–32. doi:10.1145/3439720

Zhang, X., Lee, S. K., Maeng, H., and Hahn, S. (2023). Effects of failure types on trust repairs in human–robot interactions. *International Journal of Social Robotics* 15, 1619–1635. doi:10.1007/s12369-023-01059-0
