# Supplementary material for: Human-in-the-loop error detection in an object organization task with a social robot
Source: Front Robot AI. 2024 Apr 16;11:1356827. doi: 10.3389/frobt.2024.1356827 (PMC11058786; doi:10.3389/frobt.2024.1356827)
Supplement: Supplementary file 4 [file Table3.pdf]

Table S3. Interview questions after the participant interacted with the system in all three conditions

1. How was it for you?
2. Which version did you prefer?
   - Version A (the robot speaks out the object positions)
   - Version B (the robot shows the object locations visually on the tablet)
   - Version C (the robot shows the object locations visually on the tablet and speaks out the object positions)
3. Why?
4. How did the robot learn the positions of the objects? Please tell more about how you understood the way the robot functioned. There are no right or wrong answers, please convey your impression.
5. Did you have a specific reason for the way you organized the objects?
6. Did you notice anything unexpected during the interaction with the robot?
7. Do you have any suggestions for improvement?
8. What do you think this experiment was about?
9. Was anything in the instructions unclear?
10. Is there anything else that you would like to communicate to the researchers?
